# Sex-Related Outcomes Following Drug Balloon Angioplasty in Patients from the BIOLUX P-III Registry: A Subgroup Analysis

**DOI:** 10.1007/s00270-022-03135-w

**Published:** 2022-04-20

**Authors:** Ian Patrick Barry, Reane Macarulay, Marianne Brodmann, Thomas Zeller, Matej Moscovic, Johannes Dahm, Nicola Troisi, Gunnar Tepe, Jacqueline Wong, Bibombe Patrice Mwipatayi, on behalf of the BIOLUX P-III Global Registry Investigators

**Affiliations:** 1 Department of Vascular Surgery, Royal Perth Hospital, Level 2, MRF Building, Perth, 6000 Australia; 2 Department of Angiology, Medical University Graz, Graz, Austria; 3 Clinic Cardiology and Angiology II, Universitäts-Herzzentrum Freiburg, Freiburg, Germany; 4 Department of Angiology, Institute of Cardiovascular Diseases, Kosice, Slovakia; 5 Department of Angiology and Cardiology, Herz- und Gefäßzentrum Neu-Bethlehem, Göttingen, Germany; 6 Unit of Vascular and Endovascular Surgery, Department of Surgery, San Giovanni di Dio Hospital, Florence, Italy; 7 Department of Diagnostic and Interventional Radiology, Klinikum Rosenheim, Rosenheim, Germany; 8 School of Surgery, University of Western Australia, Perth, Australia

**Keywords:** Peripheral artery disease, Diabetes, Drug-coated balloon, Drug-eluting balloon, Sex, Gender

## Abstract

**Purpose:**

To evaluate the use of drug-coated balloons in a real-world patient population with peripheral arterial disease and analyse the impact of sex on mid-term outcomes following their utilisation.

**Methods:**

The BIOLUX P-III is a prospective, international, multi-centre, registry of patients with infra-inguinal lesions treated using the Passeo-18 Lux, a drug-coated balloon. Our study is a 24-month subgroup analysis of these patients; primary endpoints were freedom from major adverse events and clinically driven target lesion re-vascularisation within 12 months post-intervention.

**Results:**

Of the 877 patients in the registry, 561 (64.0%) were male and 316 (36.0%) were female. Chronic limb threatening ischaemia (Rutherford class ≥ 4) occurred in 35.7% of males and 40.6% of females. Rates of freedom from major adverse events and clinically driven target lesion re-vascularisation at 12 months were 87.3% (95% confidence interval [CI] 84.2–89.9) and 90.4% (95% CI 86.5–93.3), and 92.3% (95% CI 89.9–94.1) and 92.9% (95% CI 89.7–95.1) in males and females, respectively. All-cause mortality at 24 months was 12.0% (95% CI 9.4–15.3) in males and 11.9% (95% CI 8.6–16.5) in females. The major target limb amputation rate at 24 months was 9.1% (95% CI 6.9–11.9) in males and 4.0% (95% CI 2.3–7.0) in females.

**Conclusion:**

Treatment with the Passeo-18 Lux DCB demonstrated high efficacy and low complication rates. Despite the greater proportion of chronic limb threatening ischaemia observed in females, males were at a greater risk of ipsilateral major limb amputation and major adverse events following drug-coated balloon utilisation.

**Clinical Trial Registration:**

NCT02276313.

**Level of Evidence:**

Level 4.

**Supplementary Information:**

The online version contains supplementary material available at 10.1007/s00270-022-03135-w.

## Introduction

The prevalence of peripheral arterial disease (PAD) is increasing [1]; however, its recognition remains lacking. While the total population burden of PAD appears to be higher in females, they are currently under-represented within prospective studies [2], leading to a paucity of sex-directed management strategies for PAD. Whether sex-related differences in presentation—such as smaller vessel diameters and increased likelihood of tibial occlusive disease in females—should be associated with different endovascular treatment modalities (i.e. balloon angioplasty vs. stenting) remains unknown [3, 4].

The role of drug-coated balloons (DCBs), such as the Passeo-18 Lux DCB (Biotronik, Berlin, Germany), has previously been outlined in trials such as the BIOLUX P-I and P-II [5–7]. Still, despite demonstrating superiority in treating symptomatic femoropopliteal lesions, there remains a concern regarding their effect in females [3]. There has been an under-representation of females in studies while they have lacked a specific gender-based endpoint. The majority of data is obtained from post hoc analysis, such as the LEVANT II study, which failed to identify a treatment effect in females [8]. However, a recent follow-up of the PTA THUNDER trial highlighted that the target lesion re-vascularisation (TLR) rate in females treated with DCBs was twice that of males [9]. Despite higher reintervention rates in females, a recent study has highlighted that males with PAD have an increased risk of mortality and major adverse cardiovascular events (MACEs) following endovascular re-vascularisation [10].

Given the previously identified differences in both presentation and outcomes following re-vascularisation, we aimed to study sex differences following DCB (the Passeo-18 Lux) utilisation in the BIOLUX P-III registry and its impact on the TLR and re-stenosis or occlusion rates. Patient data were previously published [11–13].

## Methods

### Study Design and Population

The BIOLUX P-III is a prospective, international, all-comers registry—including 44 centres across Asia, Australia, and Europe—that aimed to confirm the safety and effectiveness of the Passeo-18 Lux DCB for atherosclerotic disease of the infra-inguinal arteries; 877 patients were included. The study design was previously reported [11–13]; adults with infra-inguinal lesions suitable for endovascular therapy with the Passeo-18 Lux DCB were included. Exclusion criteria were a life expectancy of < 1 year, participation in another clinical trial, pregnancy, and failure to successfully cross the target lesion with a guidewire. Follow-up was initially planned for 24 months; however, due to the controversy regarding elevated mortality rates associated with the use of paclitaxel [14, 15], follow-up was extended to 5 years to collect mortality data.

The study was conducted according to the principles of the Declaration of Helsinki (ISO14155:2011) and local regulations and was approved by the respective independent ethics committees. All patients provided written informed consent. Endpoint-related data were monitored in at least 25% of patients. A clinical events committee adjudicated all major adverse events (MAEs), TLRs, and deaths. The trial is registered at ClinicalTrials.gov: NCT02276313.

### Study Device

The Passeo-18 Lux DCB (diameter: 2.0–7.0 mm, length: 40–120 mm) is built on the base of the Passeo-18 percutaneous transluminal angioplasty catheter. Incorporation of paclitaxel into the delivery matrix of Butyryl-tri-hexyl-citrate binds the drug into a microcrystalline structure, improving vessel wall infiltration. A sheath protects the balloon, maintaining its profile and drug coating, and is used as an insertion aid when the catheter is advanced through the introducer sheath. The DCB was used following the manufacturer’s instructions according to standard clinical practice.

### Definitions and Outcome Measure

Technical success was defined as successful completion of the endovascular procedure combined with immediate morphological success (≤ 50% reduction in residual diameter of the treated lesion), determined using visual estimation. Device success was defined as the successful delivery, inflation, deflation, and retrieval of the DCB; procedural success was defined as technical and device success without any MAE during hospital stay. Primary patency was defined as freedom from CD-TLR or restenosis, determined using a duplex ultrasound (not mandated for all patients) peak systolic velocity ratio of ≤ 2.5. MAEs were defined as device- and procedure-related mortality within 30 days, and major target limb amputation and clinically driven TLR (CD-TLR) at 6, 12, and 24 months post-index procedure. CD-TLR was defined as reintervention for stenosis (> 50% diameter) after documenting recurrent clinical symptoms.

The primary outcomes measures of our analysis, compared between sexes, were freedom from MAEs and CD-TLR within 12 months of intervention. The secondary outcome measures were technical and procedural success; freedom from MAEs and CD-TLR at 6 and 24 months; clinically driven target vessel re-vascularisation (CD-TVR); minor and major target limb amputation (any amputation above the ankle); and change in ankle-brachial index (ABI), Rutherford classification, and patient-reported outcomes (pain scale and walking impairment questionnaire) at 6, 12, and 24 months. The primary patency was assessed at 12 and 24 months.

### Statistical Analyses

Hypothesis-driven sample size estimation was not performed. For continuous variables, descriptive statistics included mean ± standard deviations and range; for categorical variables, the absolute and relative frequencies were calculated. Two-sided 95% confidence intervals (CIs) were calculated when appropriate. Freedom from CD-TLRs and MAEs and their individual components were estimated using Kaplan–Meier analysis, and intergroup comparisons were performed with the log-rank test. Standard errors were calculated using the Greenwood formula. Estimates were presented with 95% CIs. The Chi-squared or Fisher’s exact test was used to compare categorical or binary variables, while the Student’s *t*-test or Wilcoxon rank-sum test was utilised for continuous variables of independent samples. For ordinal data, the Cochran-Armitage test for trend was used. Follow-up comparisons were performed using the Wilcoxon signed-rank test. Statistical significance was set at *p* < 0.05; statistical calculations were performed using SAS software (version 9.4; SAS Institute, Cary, NC, USA).

## Results

**Fig. 1:**
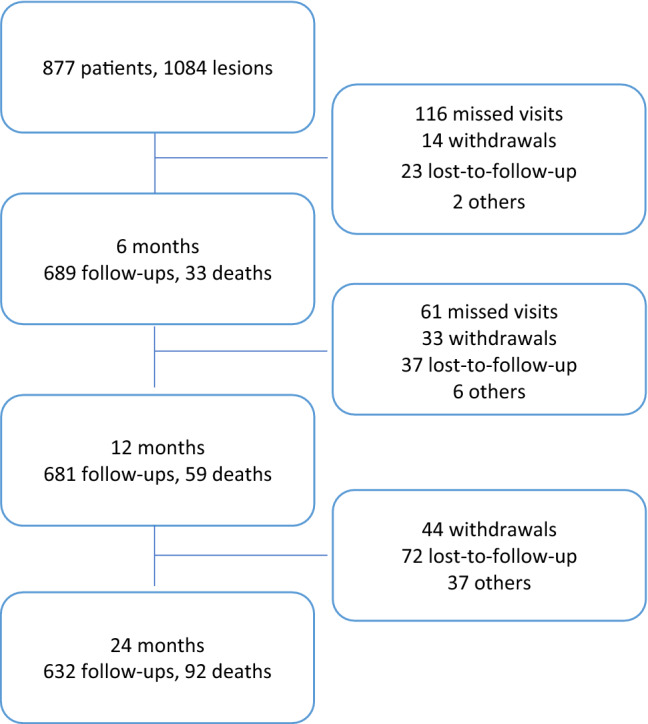
Patient disposition

Of the 877 patients in the registry, 561 (689 lesions) were male and 316 (395 lesions) were female (Fig. 1). Concomitant co-morbidities such as coronary artery disease (46.7% vs. 33.5%, *p* < 0.001) and diabetes mellitus (51.3% vs. 41.1%, *p* = 0.004) were more common in males. Conversely, hypertension (83.4% vs. 87.3%, *p* = 0.120) was more common in females; however, this lacked statistical significance (Table 1). Chronic limb threatening ischaemia (CLTI; Rutherford class ≥ 4) was present in 35.7% of males and 40.6% of females. Additionally, females were older than males during intervention and were more likely to be active smokers.

**Table 1:** Baseline demographics

	Male	Female	*p*-value
	*n* = 561	*n* = 316	
Age [years]	68.6 ± 9.8 (67.8–69.4)	72.9 ± 10.4 (71.7–74.0)	< 0.001
Body mass index [kg/m^2^]	27.1 ± 4.0 (26.7–27.4)	26.5 ± 4.8 (26.0–27.1)	0.015
Smoking history	439 (78.3%)	153 (48.4%)	< 0.001
Hypertension	468 (83.4%)	276 (87.3%)	0.120
Hyperlipidaemia	382 (68.1%)	206 (65.2%)	0.415
Diabetes	288 (51.3%)	130 (41.1%)	0.004
Renal disease (insufficiency)	188 (33.5%)	126 (39.9%)	0.059
GFR 30–60 mL/min/1.73 m^2^	82 (43.9%)	69 (54.8%)	
GFR < 30 mL/min/1.73 m^2^	38 (20.3%)	21 (16.7%)	
Dialysis	32 (17.0%)	12 (9.5%)	
Coronary artery disease	262 (46.7%)	106 (33.5%)	< 0.001
Cerebrovascular disease	114 (20.3%)	53 (16.8%)	0.206
History of PAD	336 (59.9%)	168 (53.2%)	0.053
Previous peripheral interventions	305 (54.4%)	147 (46.5%)	0.026
Cancer	64 (11.4%)	36 (11.4%)	0.993
Ankle brachial index	*n* = 266	*n* = 164	0.282
	0.67 ± 0.23 (0.64–0.70)	0.65 ± 0.24 (0.61–0.69)	
Rutherford class	*n* = 500	*n* = 279	0.013
	3.9 ± 1.3 (3.8–4.0)	3.3 ± 1.1 (3.2–3.4)	
0	0 (0%)	1 (0.3%)	
1	9 (1.6%)	3 (0.9%)	
2	97 (17.3%)	19 (6.0%)	
3	194 (34.6%)	128 (40.5%)	
4	57 (10.2%)	46 (14.6%)	
5	103 (18.4%)	65 (20.6%)	
6	40 (7.1%)	17 (5.4%)	
Wong-Baker Pain scale	*n* = 436	*n* = 235	0.014
	5.3 ± 2.8 (5.0–5.5)	5.9 ± 2.6 (5.5–6.2)	

Continuous data are presented as means ± standard deviations (95% CI); categorical data are given as the counts (percentage)

GFR: glomerular filtration rate, PAD: peripheral artery disease

The superficial femoral artery was the most frequent location for lesions in males and females (54.0% vs. 54.9%), followed by the popliteal segment (18.7% vs. 25.3%) (Table 2); most lesions were de novo (55.3% vs. 51.9%). Total occlusions (22.9% vs. 28.4%) and in-stent restenosis (10.4% vs. 11.1%) were more common in females. Most lesions in both groups were calcified, while 17.6% and 11.6% comprised heavy calcification. The reference vessel diameter (RVD) was smaller in females (4.7 ± 1.2 mm. vs. 4.5 ± 0.9 mm., *p* < 0.001), who also demonstrated more Transatlantic Inter-Society Consensus (TASC) D lesions, lesions > 200 mm in length, and active ulcerations during intervention than males.

**Table 2:** Baseline lesion characteristics

Description	Males	Females	*p*-value
	*n* = 561	*n* = 316	
Mean target lesion length [mm]	86.9 ± 74.6 (81.4–92.6)	92.5 ± 81.0 (84.5–100.6)	0.505
RVD [mm^2^]	4.7 ± 1.2 (4.6–4.8)	4.5 ± 0.9 (4.4–4.6)	< 0.001
Diameter stenosis [%]	86.4 ± 13.0 (85.4–87.4)	87.9 ± 12.4 (86.7–89.1)	0.062
Indication			0.145
De novo lesion	381 (55.3%)	205 (51.9%)	
Total occlusion	158 (22.9%)	112 (28.4%)	
Restenosis	78 (11.3%)	34 (8.6%)	
In-stent restenosis	72 (10.4%)	44 (11.1%)	
Calcification	*n* = 689	*n* = 395	0.225
None	172 (25.0%)	87 (22.0%)	
Mild	197 (28.6%)	143 (36.2%)	
Moderate	198 (28.7%)	118 (29.9%)	
Heavy	121 (17.6%)	46 (11.6%)	
TASC classification	*n* = 689	*n* = 395	0.540
A	247 (35.8%)	161 (40.8%)	
B	212 (30.8%)	102 (25.8%)	
C	127 (18.4%)	72 (18.2%)	
D	94 (13.6%)	56 (14.2%)	
Target lesion location			0.003
Common femoral	6 (0.9%)	5 (1.3%)	
Superficial femoral artery	372 (54.0%)	217 (54.9%)	
Popliteal artery	129 (18.7%)	100 (25.3%)	
Anterior tibial artery	52 (7.5%)	11 (2.8%)	
Posterior tibial artery	37 (5.4%)	9 (2.3%)	
Tibio-peroneal trunk	26 (3.8%)	14 (3.5%)	
Peroneal artery	25 (3.6%)	11 (2.8%)	
Other^a^	42 (6.1%)	28 (7.1%)	
Thrombus present	48 (7.0%)	27 (6.8%)	0.943
Lesion morphology	*n* = 689	*n* = 395	0.800
Solid lesion	338 (49.1%)	190 (48.1%)	
Diffuse lesion	348 (50.5%)	202 (51.1%)	
Number of lesions per patient	1.2 ± 0.4 (1.1–1.2)	1.2 ± 0.5 (1.1–1.2)	0.702
Amputation status target limb	*n* = 567	*n* = 318	0.029
None	521 (91.9%)	306 (96.2%)	
Minor	42 (7.4%)	12 (3.8%)	
Major	4 (0.7%)	0 (0.0%)	
Ulceration type target limb	*n* = 567	*n* = 318	
None	416 (73.4%)	227 (71.4%)	0.843
Arterial	127 (22.4%)	76 (23.9%)	
Venous	2 (0.4%)	2 (0.6%)	
Diabetic/ pressure	22 (3.9%)	13 (4.1%)	

Continuous data are presented as the means ± standard deviation (95% CI), range; categorical data are given as counts (percentage)

*CTO* chronic total occlusion, *RVD* reference vessel diameter

^a^Consisting of iliac lesions, bypass, and lesions that include more than one vessel

**Fig. 2:**
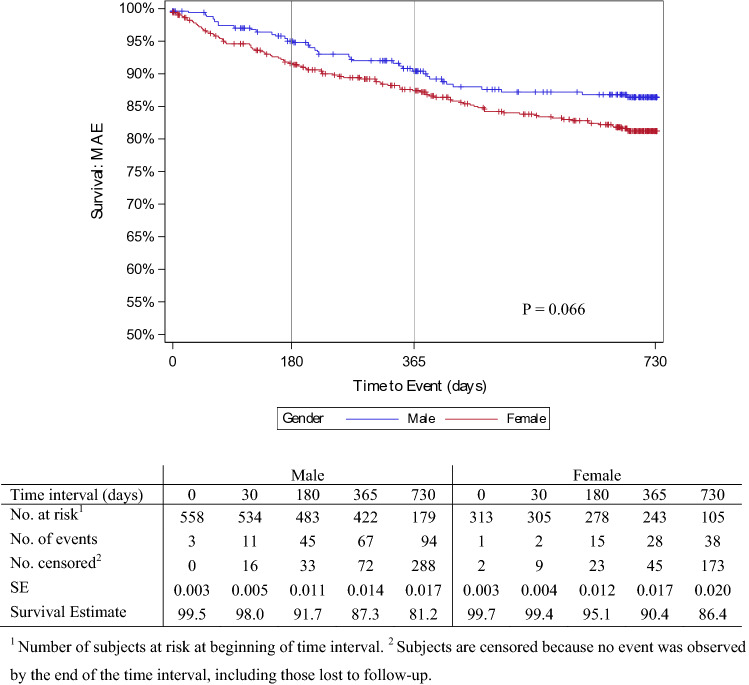
Proportion of men and women free from major adverse event (MAE) post procedure

**Fig. 3:**
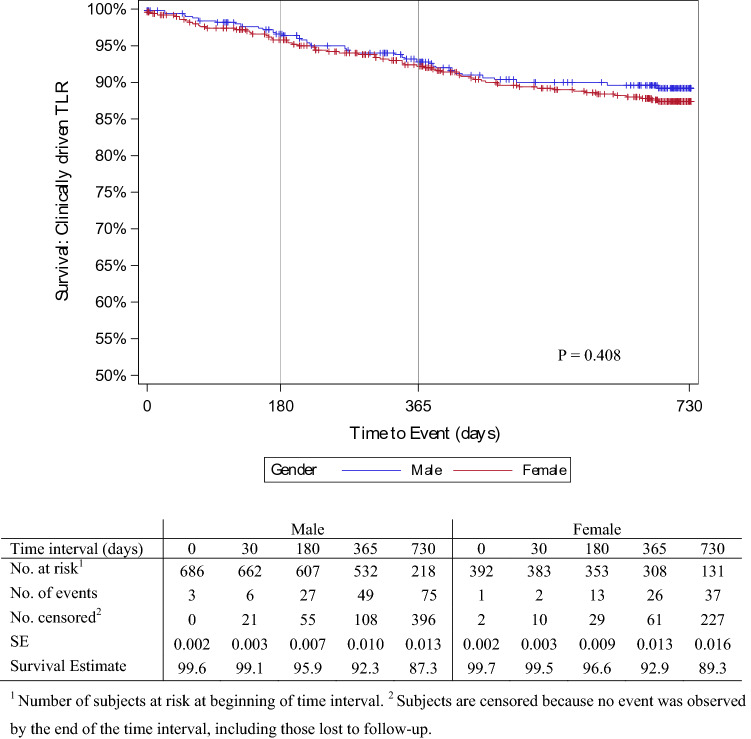
Proportion of men and women free from clinically driven TLR post procedure

The device was successfully deployed in 99.8% of cases, with few lesions in both groups requiring adjunctive bailout procedures after DCB application with a stent (16.8% vs. 13.7%) (Table 3). The clinical events committee (CEC) adjudicated rates of freedom from MAEs in males and females of 91.7% and 95.1% at 6 months; 87.3% and 90.4% at 12 months; and 81.2% and 86.4% at 24 months, respectively (*p* = 0.066) (Fig. 2). Six, 12, and 24 months post-procedure, 95.9%, 92.3%, and 87.3% of males, and 96.6%, 92.9% and 89.3% of females remained free from CD-TLR, respectively (*p* = 0.408) (Fig. 3).

**Table 3:** Procedural characteristics

Description	Males	Females	p-value
	*n* = 561	*n* = 316	
Target lesion preparation	496 (72.0%)	296 (74.9%)	0.292
Uncoated balloon	481 (83.9%)	362 (82.6%)	
Rotational thrombectomy	17 (3.0%)	11 (3.2%)	
Atherectomy	16 (2.8%)	13 (3.8%)	
Scoring balloon	21 (3.7%)	6 (1.7%)	
Cutting balloon	21 (3.7%)	8 (2.3%)	
Other	8 (1.4%)	4 (1.2%)	
Device diameter [mm]	4.1 ± 1.2 (4.0–4.2)	3.8 ± 1.0 (3.7–3.9)	< 0.001
Device length [mm]	77.7 ± 52.5 (73.3–82.2)	82.1 ± 51.5 (76.4–87.8)	0.096
Passeo-18 Lux diameter [mm^2^]	4.7 ± 1.2 (4.6–4.7)	4.5 ± 1.0 (4.5–4.6)	0.005
Passeo-18 Lux length [mm]	88.5 ± 32.3 (86.4–90.6)	88.4 ± 32.3 (86.7–92.2)	0.562
Paclitaxel dose per patient [mg]	*n* = 561	*n* = 316	0.720
	7.3 ± 5.6 (6.9–7.8)	7.6 ± 5.7 (6.9–8.2)	
Maximum pressure applied [atm]	8. 9 ± 2. 9 (8.7–9.1)	8.5 ± 2. 8 (8.2–8.7)	0.041
Cumulative inflation time [s]	144 ± 62 (140–148)	149 ± 63 (143–154)	0.094
Treatment with additional device	173 (25.1%)	92 (23.3%)	0.503
Uncoated balloon	78 (32.6%)	56 (41.8%)	
Drug-coated balloon	6 (2.5%)	8 (6.0%)	
Stent	141 (59.0%)	62 (46.3%)	
Other	14 (5.9%)	8 (6.0%)	
Device success^a^	*n* = 917	*n* = 534	> 0.999
	915 (99.8%)	533 (99.8%)	
Technical success^b^	*n* = 689	*n* = 568	0.449
	680 (98.7%)	387 (98.0%)	
Procedural success patients^c^	*n* = 561	*n* = 316	> 0.999
	542 (96.6%)	305 (96.5%)	

Continuous data are presented as the means ± standard deviation (95% CI), range; categorical data are given as the counts (percentage)

^a^Device success: successful delivery, inflation, deflation, and retrieval of Passeo-18 Lux

^b^Technical success: residual diameter reduction of the treated lesion as determined by visual estimation ≤ 50%

^c^Procedural success: technical and device success without the occurrence of any major adverse events

**Fig. 4:**
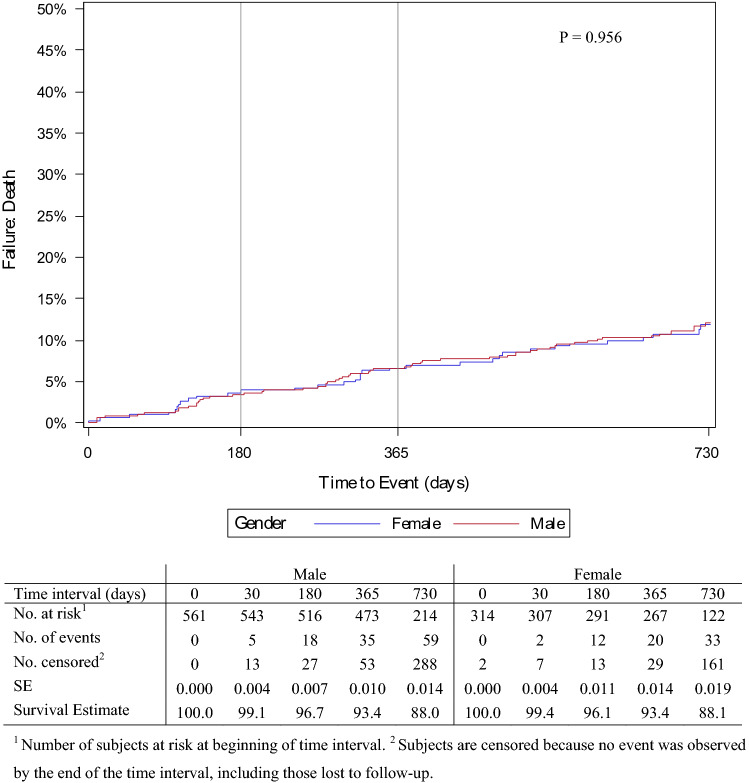
Proportion of men and women free from all-cause mortality post procedure

**Fig. 5:**
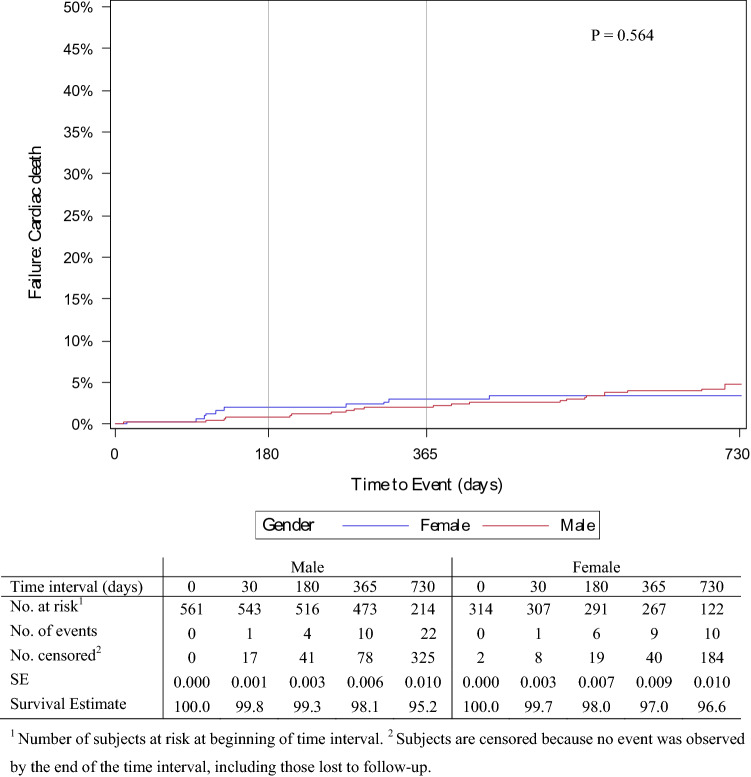
Proportion of men and women free from cardiac-related mortality post procedure

**Fig. 6:**
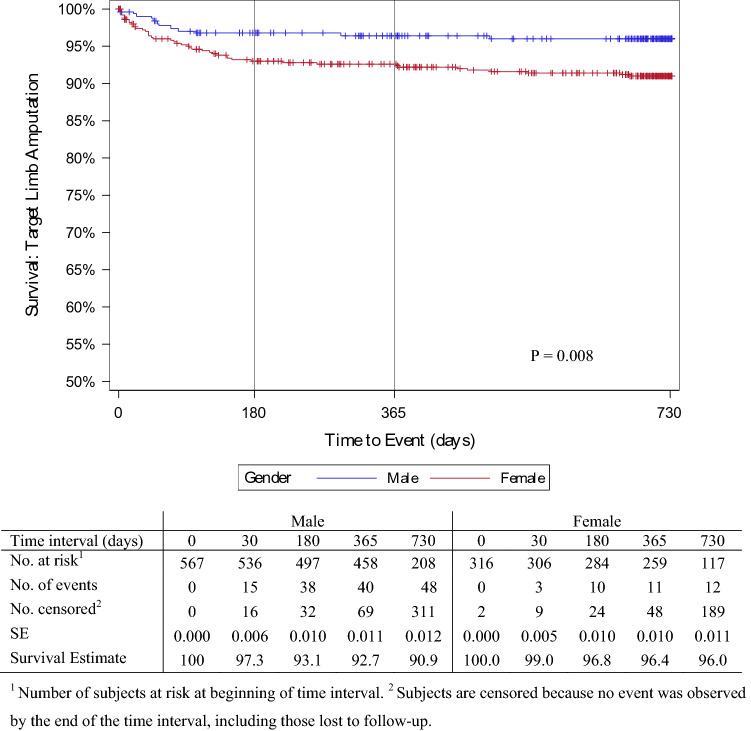
Proportion of men and women free from target limb amputation post procedure

All-cause mortality at 24 months was 12.0% in males and 11.9% in females (Fig. 4). Death was most common between 12 and 24 months post-procedure for both sexes; however, this was not significant (*p* = 0.956). Cardiac-related mortality (Fig. 5) was also relatively similar at 24 months between males and females (4.8% [95% CI 3.2–7.3] vs. 3.4% [95% CI 1.8–6.2], *p* = 0.565). Figure 6 shows the overall amputation rate over time; the log-rank test indicated a significant difference in the hazard ratio between the sexes (*p* = 0.009). Overall amputations were most common 6 months post-procedure, regardless of sex (38 males and 10 females). Twenty-four months post-procedure, the overall target limb amputation rate was 9.1% (95% CI 6.9–11.9) in males and 4.0% (95% CI 2.3–7.0) in females; the log-rank test revealed that the major target limb amputation rate was significantly higher (*p* = 0.004) in males than females (4.9% vs. 1.1%, respectively) (Supplementary Fig. 1).

Mean ABI improvements 24 months post-procedure were 0.22 ± 0.27 in males and 0.18 ± 0.26 in females (males: 0.67 ± 0.23–0.87 ± 0.22, females: 0.65 ± 0.24–0.86 ± 0.22) (Supplementary Fig. 2). Most patients improved by at least one Rutherford class in both groups (males: − 2.19 ± 1.67, females: − 2.50 ± 1.62) within 24 months (Supplementary Fig. 3). Sustained improvements in pain, reported via the pain scale and walking impairment questionnaire, were observed in both groups at 24 months (75.2% in males vs. 75.9% in females) (Supplementary Fig. 4).

## Discussion

Our analysis demonstrated that 36% of participants within the BIOLUX P-III registry were female. Sex-based differences were observed, with females more commonly exhibiting CLTI (Rutherford class ≥ 4) during intervention, and males exhibiting a significantly higher rate of major target limb amputation following re-vascularisation. These findings re-emphasise the possible role of sex-based management strategies in PAD.

Several previously identified factors were proposed to explain the role of sex in PAD. First, there is a significant, sex-based difference in symptomatology; in a previous study, 9% of males and 13% of females with PAD were asymptomatic [16]. This was difficult to establish within our analysis, as asymptomatic status was an exclusion criterion. The varying presentations of atypical symptoms during assessment and subsequent delayed diagnosis and intervention in females is likely why previous studies primarily involved male participants; thus, females were recurrently under-represented. This was previously highlighted by the Walking and Leg Circulation Study cohort, which demonstrated that females were twice as likely to have atypical exertional leg symptoms when compared with males [17, 18]. Delayed diagnosis and intervention in females with PAD was also outlined within our analysis, with more females exhibiting CLTI (40.6% vs. 35.7%), and a significantly higher mean Rutherford classification (*p* = 0.013) during intervention.

There also appears to be several anatomical variations between sexes, with smaller vessel diameters and a more significant burden of occlusive disease in females [3]. In our analysis, occlusive disease was present in 28.4% of females and 22.9% of males. RVD was found to be significantly smaller in females (*p* < 0.001); correspondingly, the mean target lesion length was greater in females, although this lacked significance (*p* = 0.505). Despite these variations in symptomatology and underlying anatomy, the pathogenesis is believed to be comparable between sexes. The process involves vascular inflammation, decreased nitric oxide levels, increased vascular smooth muscle cell migration, and proliferation [4]; the paclitaxel coating utilised on the Passeo-18 Lux DCB aims to target some of these mechanisms to reduce the rate of neointimal hyperplasia and associated restenosis [19].

Another previously identified sex-based difference is that males with PAD have more co-morbidities than females [2]. In our analyses, coronary artery disease, diabetes mellitus, cerebrovascular disease, and dialysis dependence were more prevalent in males. Previously, this significant cardiovascular burden was identified as a major independent contributor to both overall and cardiac mortality. A recent study highlighted the propensity of males towards MACEs in such a setting [10]; interestingly, our study did not identify significant differences in overall (12.0% vs. 11.9%, *p* = 0.956) or cardiac-related (4.8% vs. 3.4%, *p* = 0.565) mortality.

Concerns regarding DCB treatment effects in females—specifically regarding TLR—arose following the PTA THUNDER trial [9], highlighting that females were twice as likely to require TLR following DCB utilisation when compared with males. In our analysis, females exhibited more advanced arterial disease at intervention (Rutherford class ≥ 4); however, clinically driven TLR was more common in males at 24 months. MAEs were also slightly more common in males, indicating that this concern may warrant further investigation and be device specific.

Males with PAD previously demonstrated a higher risk of target limb amputation [20, 21]; in our study, rates were 9.1% in males and 4.0% in females at 24 months. The reasons for this may be multifactorial. One proposed explanation is that males may not observe their feet as often as females with a subsequent delay in medical review [22]; however, this contradicts the findings within our analysis of females presenting at a later stage (CLTI: 40.6% vs. 35.7%). In our study, this increased risk of lower extremity amputation in males was further emphasised by their significantly higher rate of major target limb amputation (*p* = 0.004).

Rutherford classification, ABI, and pain score all significantly improved at 6, 12, and 24 months, with Rutherford classification improvements in 79.9% of males and 84.8% of females at 24 months. ABI also improved in both males (0.22 ± 0.27) and females (0.18 ± 0.26), and pain scores fell from 5.3 to 2.0 ± 2.4 in males, and 5.9 to 2.5 ± 2.6 in females during follow-up. These substantial improvements were particularly encouraging in females, as more aggressive and extensive disease status were suggested at intervention.

## Limitations

The BIOLUX P-III is an all-comers registry with many limitations. First, it is prone to selection bias; since physicians enrolled patients for whom they believed therapy would be appropriate, the decision to enrol was not based on randomisation or sequential patient identification. Therefore, crural vessel disease was under-represented, reflecting the reluctance of practitioners to use DCBs for infra-popliteal lesions. Inability to cross the lesion—either primarily or via another adjunctive technique per the clinician’s discretion—resulted in exclusion from the registry, further contributing to selection bias. As this criterion was not the primary intention of the registry, this data was not captured.

Loss to follow-up was a further limitation, while the exclusion of patients with a life expectancy of < 1 year may have influenced mortality rates. No wound staging classification systems were utilised to assess the risk of amputation; such data on wound healing would have been informative for long-term prognosis. Additionally, although medication use substantially impacts outcomes, this was not formally assessed within our study; patients with PAD typically require control of optimal glucose level [23, 24], hypertension, and dyslipidaemia, with concomitant antiplatelet therapy [23].

The study was not initially designed to analyse all patient subgroups; thus, further enrolment of patients was not based on rigorous sample size calculation. Additionally, performance outcomes such as ABI, Rutherford classification assessments, or duplex ultrasound, were not available for all patients. Finally, this study did not include core laboratory evaluations, such as intraoperative angiography and/or follow-up duplex ultrasound, that may have offered meticulous quality control of our results.

## Conclusion

Treatment with the Passeo-18 Lux DCB resulted in high efficacy and low complication rates. Despite there being more females with CLTI, males appeared to be associated with poorer outcomes, demonstrating a greater risk of ipsilateral major limb amputation and MAEs following DCB utilisation.

## Supplementary Information

Below is the link to the electronic supplementary material.

Supplementary file1 (DOCX 30 KB)

Supplementary file2 (DOCX 26 KB)

Supplementary file3 (DOCX 25 KB)

Supplementary file4 (DOCX 25 KB)
