# Supplementary material for: Sex-Related Outcomes Following Drug Balloon Angioplasty in Patients from the BIOLUX P-III Registry: A Subgroup Analysis
Source: Cardiovasc Intervent Radiol. 2022 Apr 20;45(7):918–28. doi: 10.1007/s00270-022-03135-w (PMC9225976; doi:10.1007/s00270-022-03135-w)
Supplement: Supplementary file 1 — Supplementary file1 (DOCX 30 KB) [file 270_2022_3135_MOESM1_ESM.docx]

P = 0.003

|  | Male | | | | | Female | | | | |
| --- | --- | --- | --- | --- | --- | --- | --- | --- | --- | --- |
| Time interval (days) | 0 | 30 | 180 | 365 | 730 | 0 | 30 | 180 | 365 | 730 |
| No. at risk^1^ | 567 | 544 | 511 | 468 | 214 | 316 | 309 | 292 | 268 | 122 |
| No. of events | 0 | 6 | 22 | 24 | 26 | 0 | 0 | 2 | 2 | 3 |
| No. censored^2^ | 0 | 17 | 34 | 75 | 327 | 2 | 9 | 24 | 48 | 193 |
| SE | 0.000 | 0.004 | 0.008 | 0.008 | 0.009 | 0.000 | 0.000 | 0.004 | 0.004 | 0.006 |
| Survival Estimate | 100.0 | 98.9 | 96.0 | 95.6 | 95.1 | 100.0 | 100.0 | 99.3 | 99.3 | 98.9 |

^1^ Number of subjects at risk at beginning of time interval. ^2^ Subjects are censored because no event was observed by the end of the time interval, including those lost to follow-up.

**Supplementary Figure 1.** Proportion of men and women free from major target limb amputation post procedure
