# Supplementary material for: Sex-Related Outcomes Following Drug Balloon Angioplasty in Patients from the BIOLUX P-III Registry: A Subgroup Analysis
Source: Cardiovasc Intervent Radiol. 2022 Apr 20;45(7):918–28. doi: 10.1007/s00270-022-03135-w (PMC9225976; doi:10.1007/s00270-022-03135-w)
Supplement: Supplementary file 2 — Supplementary file2 (DOCX 26 KB) [file 270_2022_3135_MOESM2_ESM.docx]

**Supplementary Figure 2.** Change in mean ABI between baseline and follow-up
