# Supplementary material for: Sex-Related Outcomes Following Drug Balloon Angioplasty in Patients from the BIOLUX P-III Registry: A Subgroup Analysis
Source: Cardiovasc Intervent Radiol. 2022 Apr 20;45(7):918–28. doi: 10.1007/s00270-022-03135-w (PMC9225976; doi:10.1007/s00270-022-03135-w)
Supplement: Supplementary file 3 — Supplementary file3 (DOCX 25 KB) [file 270_2022_3135_MOESM3_ESM.docx]

**Supplementary Figure 3.** Change in mean Rutherford class between baseline and follow-up.
